# Supplementary material for: Chromothripsis during telomere crisis is independent of NHEJ, and consistent with a replicative origin
Source: Genome Res. 2019 May;29(5):737–49. doi: 10.1101/gr.240705.118 (PMC6499312; doi:10.1101/gr.240705.118)
Supplement: Supplemental Material [file supp_gr.240705.118_Supplemental_file_1.zip › contigs/annotated_contigs/DB54/contig.2.DB54_length_535_mean_cov_8.69158878505.docx]

**DB54_length_535_mean_cov_8.69158878505**

```
TCTGTCCACACGTGGCACCTATGTGCTGTGAGAGATACAAGCACTCTTTGTGTAAGCAAAAGCATAGCATTGCTTGAGTTTTATTATAG
 >chr6:155406955-155407173 + E=1e-119
ATATAGTTTACCTAGCACCACCTTGGATCTCATAGACTCACATACTCAAGGTGCCCTGTTTGATCTTTCTGTGGTCACAAGATATTGCA

GTCCCGGCCCACTGCAAATTCTCCCTTAAAATGGGACTTT|ATTATGGGGGCGGTTTCCCCCATGCTGTTCTCATGATAGTGAGTTCTC
                                          >chr6:155515983-155516300 + E=3e-179
ATTAGATCTGATCATTTTATAAGGGGCTTCCCCCTTTGCTGGGCACTGATTCTTCTTGCTGCCACCATGTGAAGAAAGACATGTTTGCG

TCCCCTTCCACCATGATTGTAAGTTTCCTGAGGCCTCCGCAGCCCTGCGGAACTGTGAGTCAATGAAACCTCTTTATTTTATCTATTAC

CCAGTCTCAGGTATGTCTTTATTAGCAGTGTGAGAATGGATTAATACAAACATTAACTTTATTTTTCAACAAGATTCTAAGGCTTAAGG

TG
```
